# Case Report: Neuromyelitis Optica After Treatment of Uveal Melanoma With Nivolumab and Ipilimumab

**DOI:** 10.3389/fonc.2022.806501

**Published:** 2022-03-23

**Authors:** Karima Khimani, Sapna P. Patel, Andrew Whyte, Nagham Al-Zubidi

**Affiliations:** ^1^ Department of Ophthalmology, University of Texas Medical Branch, Galveston, TX, United States; ^2^ Department of Melanoma Medical Oncology, The University of Texas MD Anderson Cancer Center, Houston, TX, United States; ^3^ Department of Head and Neck Surgery, Section of Ophthalmology, The University of Texas MD Anderson Cancer Center, Houston, TX, United States; ^4^ Department of Ophthalmology, Blanton Eye Institute, Houston Methodist Hospital, Houston, TX, United States; ^5^ Department of Ophthalmology, Weill Cornell Medicine, New York, NY, United States

**Keywords:** neuromyelitis optica spectrum disorder, NMO, immune checkpoint inhibitor, ipilimumab, nivolumab, uveal melanoma

## Abstract

The development of immune checkpoint inhibitors (ICIs) has greatly improved survival of patients with advanced malignancies. ICIs can cause immune-related adverse effects (irAEs) involving any organ. Neurological irAEs are infrequent and have mostly been reported in patients with melanoma. We describe the case of a 57-year-old male with right eye uveal melanoma, gene expression profile (class 2), and PRAME (preferentially expressed antigen in melanoma) positivity, who received plaque brachytherapy with Iodine-125 for 4 days with subsequent adjuvant ICIs (immune checkpoint inhibitors), nivolumab and ipilimumab. 18 weeks after discontinuation of immunotherapy, the patient presented with acute onset of left-sided headaches, pain with eye movements, and vision loss. The patient was tested positive for serum anti-aquaporin-4 antibody (AQP4-Ab) and was diagnosed with neuromyelitis optica spectrum disorder (NMOSD). Subsequently, he was treated with 5 days of intravenous methylprednisolone followed by an oral prednisone taper over 10 weeks, with improvement in symptoms. We report a unique case of neuromyelitis optica spectrum disorder (NMOSD) following treatment with ICIs. To our best knowledge, this is the third reported case in English literature of NMOSD following ICI therapy and the first reported case of NMOSD caused by ICI treatment in uveal melanoma.

## Introduction

The development of immune checkpoint inhibitors (ICIs) has greatly improved survival of patients with advanced malignancies such as melanomas and non-small-cell lung cancers over the last decade (1–11). The cytotoxic T lymphocyte antigen 4 (CTLA-4), programmed cell death 1 (PD-1) surface protein and programmed cell death 1 ligand 1 (PD-L1) are immune checkpoints that inhibit T-cell effector mechanisms and reduce immune responses against tumor cells (12). ICIs such as ipilimumab (CTLA-4 inhibitor), nivolumab (PD-1 inhibitor), and pembrolizumab (PD-1 inhibitor) block these interactions between checkpoints and their ligands with a resultant increase in T-cell activation, thereby enhancing the antitumor immune response (12). A consequence of the augmented T-cell response is a spectrum of immune-related adverse effects (irAEs) involving every organ particularly the skin, gastrointestinal tract, liver, and endocrine system (13). Neurological irAEs are infrequent and have mostly been reported in patients with melanoma (13). We report a unique case of neuromyelitis optica spectrum disorder (NMOSD) provoked by treatment with ICIs. To our best knowledge, this is the third reported case in English literature of NMOSD tempted by ICI therapy and the first reported case of NMOSD caused by ICI treatment in uveal melanoma (14, 15).

## Case Description

**Figure 1:**
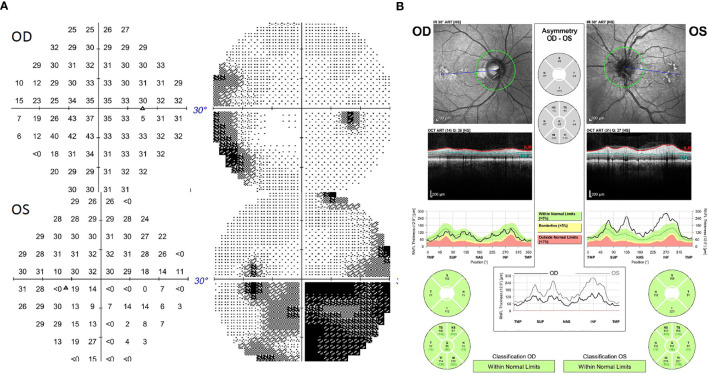
**(A)** Humphrey Visual Field (HVF) 30-2 showing an inferior altitudinal defect in the left eye. **(B)** Optical coherence tomography (OCT) of the optic nerve showing increased thickness of the peripapillary retinal nerve fiber layer in the left eye.

**Figure 2:**
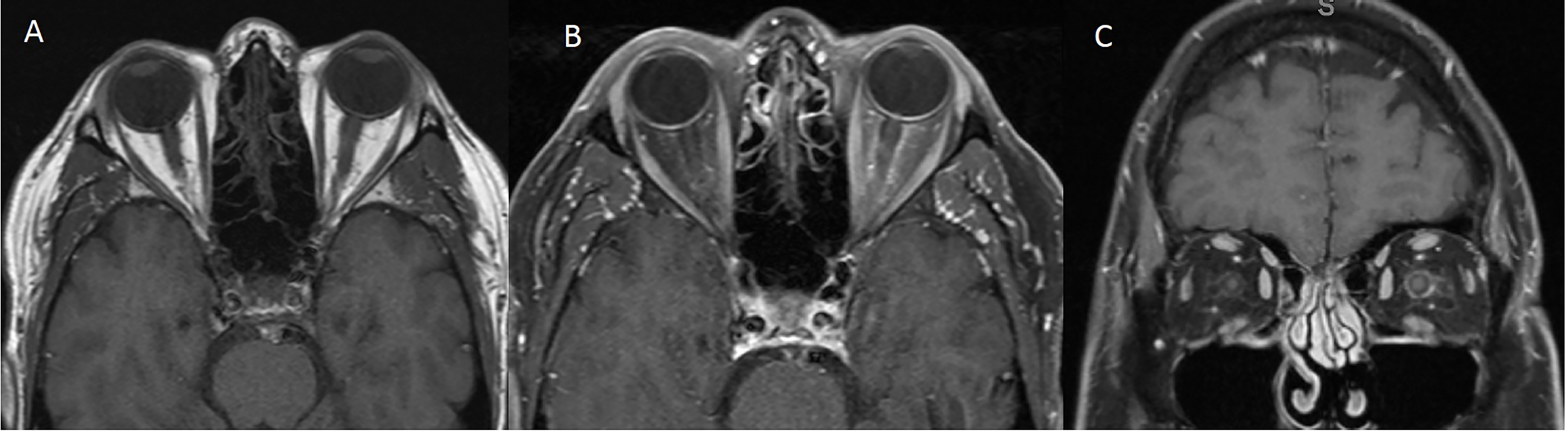
MRI of the orbits [**(A)** T1 Axial without contrast, **(B)** T1 Axial with contrast, **(C)** T1 Coronal with contrast] showing edema of the intraorbital and intracanalicular left optic nerve and associated enhancement of the intracanalicular segment with gadolinium.

The patient was a 57-year-old man with the diagnosis of right-eye uveal melanoma since 2017. His uveal melanoma had a high-risk gene expression profile (Class 2) and was positive for the cancer-testis antigen PRAME which stands for “preferentially expressed antigen in melanoma”. Primary treatment consisted of plaque brachytherapy with Iodine-125 for 4 days with subsequent adjuvant ICI, nivolumab 240 mg intravenously every 2 weeks, and ipilimumab 1 mg/kg intravenously every 6 weeks, on a clinical trial. ICIs were discontinued after 18 weeks due to development of side effects including pneumonitis, uveitis, and hypophysitis. He required high-dose steroids, mechanical ventilation, and physiologic hormone replacement of thyroid and cortisone. Three months later, the patient presented with acute onset of left-sided headaches, pain with eye movements, and vision loss. Neuro-ophthalmology examination revealed visual acuity of 20/30 in the right eye (OD) and 20/40 in the left eye (OS) with left relative afferent pupillary defect (RAPD). Color vision (Hardy–Rand–Rittler) was 5/14 OD and 4/14 OS. Intraocular pressures were within normal limits in both eyes. Slit-lamp examination was unremarkable. Extraocular motility was full, but with left-eye discomfort with eye movements. Dilated fundus exam showed circumferential optic disc edema with peripapillary hemorrhage OS and a normal disc in the OD. Humphrey visual field (HVF) 24-2 showed an inferior altitudinal defect in the left eye (**Figure 1A**). Optical coherence tomography (OCT) of the optic nerve revealed increased thickness of the peripapillary retinal nerve fiber layer OS (**Figure 1B**) consistent with left optic disc edema. T2-weighted MRI of the orbit with and without contrast revealed edema of infraorbital and intracanalicular left optic nerve and associated enhancement of the intracanalicular segment with gadolinium, suggesting optic neuritis (**Figure 2**). The serological workup for syphilis, tuberculosis, lupus, sarcoidosis, Sjogren syndrome, and myelin oligodendrocyte glycoprotein (MOG) was negative. The anti-aquaporin-4 antibody (AQP4-Ab) was detected in the serum with NMO/AQP4 titer of 1:1,000.

## Diagnostic Assessment, Intervention, and Outcome

**Figure 3:**
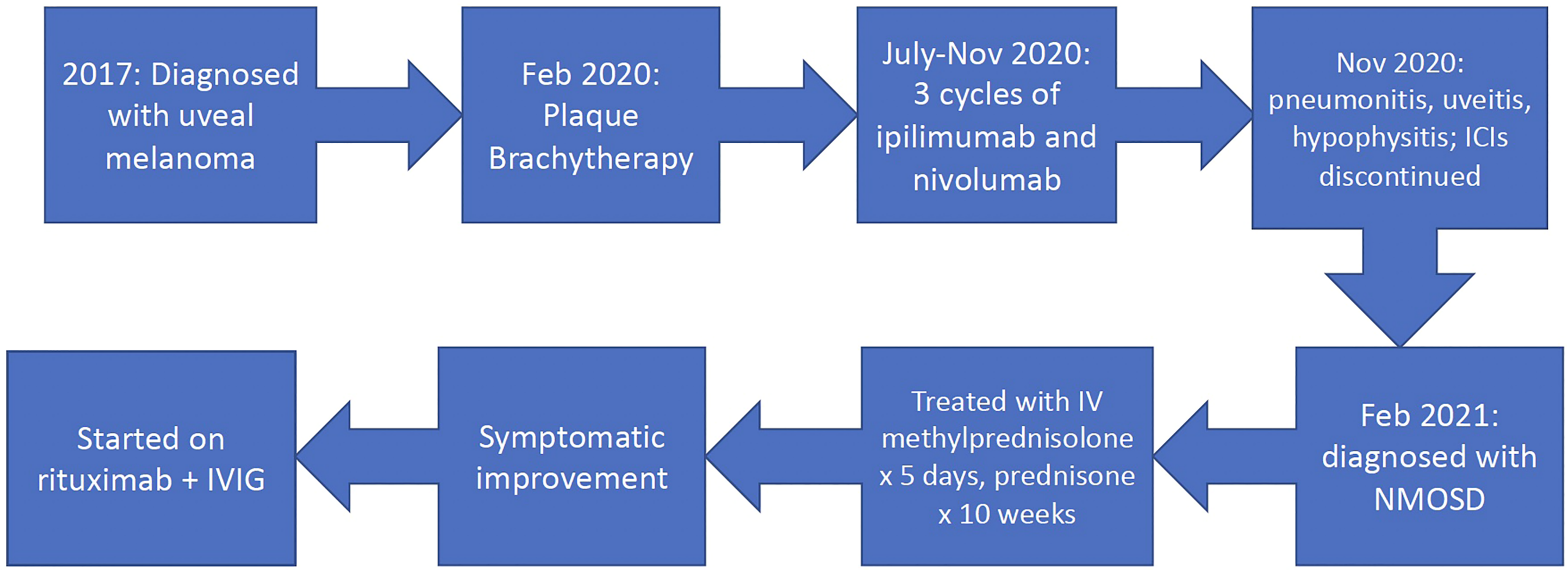
Timeline. ICIs, immune checkpoint inhibitors; NMOSD, neuromyelitis optica spectrum disorder; IVIG, intravenous immune globulin.

Based on diagnostic criteria, the patient was diagnosed with neuromyelitis optica spectrum disorder (NMOSD) and was treated with 5 days of 1,000 mg intravenous methylprednisolone followed by an oral prednisone taper over 10 weeks, with improvement in symptoms. He remained on physiologic hydrocortisone for adrenal insufficiency from hypophysitis. The patient was referred to a neurologist for further management of NMOSD. After extensive discussion regarding eculizumab vs. rituximab between medical oncology, neuro-ophthalmology, and neurology, the mutual decision was made to move forward with rituximab for treatment of this patient’s NMOSD at a dose of 1,000 mg intravenously every 6 months. Interestingly, the patient was found to have low IgG levels, and he was started on intravenous immune globulin (IVIG) along with rituximab. The etiology of low IgG levels is unknown and could be related to the ICI treatment. The timeline of his diagnoses and treatment is illustrated in **Figure 3**. The patient’s visual acuity improved to 20/30 OS after treatment.

## Discussion

Our patient developed anti-AQP4 antibody-positive NMO 3 months after receiving adjuvant treatment with the ICIs nivolumab and ipilimumab for a total of 18 weeks. NMOSD is a central nervous system (CNS) inflammatory disease spectrum that is associated with serum AQP4 immunoglobulin G (IgG) antibodies (14). The mechanism of ICIs causing NMO is not completely understood. However, in a case of NMOSD induced by treatment of non-small cell lung cancer with nivolumab, Narumi et al. suggested the activation of Type 2 helper T cells by ICIs that leads to stimulation of B cells, producing anti-AQP4 antibodies (14). On the other hand, Yshii et al. proposed three mechanisms of CNS inflammation induced by ICI therapy (13). First, ICIs could stimulate an autoimmune response that may cross-react with CNS antigens (13). This mechanism supports a paraneoplastic process. Second, ICIs could stimulate tumor-directed and CNS-directed immune responses independently (13). Third, ICIs could recognize their respective CTLA4, PD-1, or PD-L1 receptors on cells of the CNS such as neurons, astrocytes, and endothelial cells, thereby directly inducing a complement-mediated or cell-mediated cytotoxic inflammatory reaction (13). In metastatic cutaneous melanoma treated with ICI, the assumption is to have a combination of T-cell activation and autoimmune reaction against a mutation in the gene encoding N-methyl-d-aspartate receptor (NMDAR) (13). NMOSD induced by ICI treatment in uveal melanoma may have a similar combined mechanism of B cell-mediated immune response and autoimmune reaction against CNS antigens. Subsequently, neuronal counterparts can be affected resulting in neurological manifestations (13). ICI-induced neurological irAEs include Guillain-Barre syndrome, chronic inflammatory demyelinating polyneuropathy, aseptic meningitis, limbal encephalopathy, and cerebellitis (15). Cumulatively, the incidence of neurological irAEs from ipilimumab, nivolumab, and pembrolizumab has been reported to be less than 1% by prior studies (16). However, Spain et al. reported a higher rate (14%) of neurotoxicity in patients treated with a combination of nivolumab and ipilimumab (16).

Our patient presented with an anti-AQP4 antibody positive NMO after 18 weeks of adjuvant nivolumab plus ipilimumab. To our knowledge, this is the third known case in English literature of NMOSD caused by ICI treatment. The first case was a 75-year-old male with squamous cell carcinoma of the lung who developed transverse myelitis and was anti-AQP4 positive after 1 month of nivolumab treatment (14). The second case was a 63-year-old Asian female with metastatic lung adenocarcinoma and developed anti-AQP4 antibody positive NMO after the first cycle of pembrolizumab (15). In our case, it is unclear if NMO is related to uveal melanoma or to the ICI treatment. Our presumed mechanism is the blockage of immune checkpoint triggering a B cell-mediated immune response in the CNS, resulting in NMO. Conversely, even though uveal melanoma has low tumor-specific mutation rates compared to cutaneous melanoma, low mutational load of uveal melanoma may still bring about T-cell activation and antitumor immune response (17). Further studies are needed to clarify the pathophysiology of NMOSD induced by ICI therapy for uveal melanoma.

## Data Availability Statement

The original contributions presented in the study are included in the article/supplementary material. Further inquiries can be directed to the corresponding author.

## Author Contributions

KK and SP contributed equally to this work and share first authorship. All authors contributed to the article and approved the submitted version.

## Conflict of Interest

Author SP received institutional clinical trial or research support from Bristol Myers Squibb, Foghorn Therapeutics, InxMed, Novartis, Provectus Biopharmaceuticals, Reata, TriSalus Life Sciences, is on the advisory board for Cardinal Health, Castle Biosciences, and TriSalus Life Sciences, and has provided consulting services for Advance Knowledge in Healthcare and Immunocore.

The remaining authors declare that the research was conducted in the absence of any commercial or financial relationships that could be construed as a potential conflict of interest.

## Publisher’s Note

All claims expressed in this article are solely those of the authors and do not necessarily represent those of their affiliated organizations, or those of the publisher, the editors and the reviewers. Any product that may be evaluated in this article, or claim that may be made by its manufacturer, is not guaranteed or endorsed by the publisher.
